# Structural and functional insights into NAD(P)H-quinone oxidoreductases in lavender: implications for abiotic stress tolerance and essential oil production

**DOI:** 10.3389/fpls.2025.1661227

**Published:** 2025-08-28

**Authors:** Dafeng Liu, Na Li, Huashui Deng, Daoqi Song, Minawaier Maimaiti, Ayidana Nuerbieke, Mingtai Yekepeng, Kailibinuer Aili

**Affiliations:** ^1^ Xinjiang Key Laboratory of Lavender Conservation and Utilization, College of Biological Sciences and Technology, Yili Normal University, Yining, Xinjiang, China; ^2^ School of Life Sciences, Xiamen University, Xiamen, Fujian, China

**Keywords:** *Lavandula angustifolia* (lavender), NAD(P)H-quinone oxidoreductase, three-dimensional (3D) structures, gene expression levels, abiotic stress

## Abstract

Lavender essential oils (EOs) are economically valuable, with biosynthesis linked to photosynthesis. NAD(P)H-quinone oxidoreductases (NDHs) play a crucial role in regulating photosynthetic processes. To better understand the functional roles and mechanisms of NDHs, we investigated *Lavandula angustifolia* NDHs (LaNDHs) using AlphaFold2 for structural prediction and RT-qPCR for expression analysis. Gene *LaNDHs* showed highest expression in leaves compared to other tissues (stems, roots and flowers), with upregulation under cadmium ion, heat, salt, and blue light. These findings suggest LaNDHs enhance stress tolerance and photosynthesis, offering potential for improving EO yield.

## Introduction

Lavender (*Lavandula angustifolia*) is an aromatic shrub cultivated for its essential oils (EOs), widely used in cosmetics and medicine (Crişan et al., 2023; de Melo Alves Silva et al., 2023; Wilson et al., 2021). The quality of lavender EOs is primarily influenced by their monoterpene composition, which predominantly features linalool, linalyl acetate, borneol, camphor, and 1,8-cineole (Prosche and Stappen, 2024; Vairinhos and Miguel, 2020; Aarshageetha et al., 2023; Liu et al., 2025b). The highest quality EOs are typically derived from the flowering tops of *Lavandula angustifolia*, often referred to as ‘true lavender,’ which is celebrated for its unique fragrance and has been highly valued since ancient times. Lavender EOs are extensively used in the cosmetics, hygiene, and alternative medicine industries (Hedayati et al., 2024; Khan et al., 2024; Li et al., 2024; Liu et al., 2025a; Guo and Wang, 2020). For example, EOs with elevated camphor content are employed in inhalants for treating respiratory conditions such as coughs and colds, as well as in liniments and balms for topical analgesic applications (Malloggi et al., 2021; Batiha et al., 2023; Braunstein and Braunstein, 2023; Liu et al., 2024). Furthermore, camphor has been investigated as a radiosensitizing agent to enhance tumor oxygenation prior to radiotherapy (Malloggi et al., 2021; Batiha et al., 2023; Braunstein and Braunstein, 2023; Liu et al., 2024).

EO biosynthesis depends on photosynthesis, which provides ATP/NADPH and carbon precursors for terpenes (Croce et al., 2024; Reece and Sharkey, 2020). Factors such as light intensity, spectrum, and photoperiod significantly affect the yield of lavender EOs by modulating key enzymes involved in the process (Evans, 2013). Optimal light conditions enhance both photosynthetic efficiency and the biosynthesis of monoterpenes (Li et al., 2023, 2025). Additionally, *Lavandula angustifolia* NAD(P)H-quinone oxidoreductases (LaNDHs) represent another important factor influencing the yield and quality of EOs (Croce et al., 2024; Reece and Sharkey, 2020; Dinkova-Kostova and Talalay, 2010). LaNDHs boost EOs’ yield and quality by reducing oxidative stress and stabilizing terpene biosynthesis. LaNDHs maintain redox balance, enhancing terpene synthase activity and precursor availability. Efficient LaNDHs function leads to higher the production of EOs and preserved aromatic compounds, improving overall characteristics of EOs. LaNDHs are cytosolic enzymes that catalyze the reduction of quinones and a broad range of other substrates (Pey et al., 2019). Cellular defense mechanisms against oxidative stress involve various protective pathways, with LaNDHs playing a central role (Dinkova-Kostova and Talalay, 2010). This enzyme catalyzes the two-electron reduction of quinones to hydroquinones, utilizing NADH or NAD(P)H as electron donors. This reaction prevents the formation of reactive semiquinone intermediates, thereby inhibiting the generation of reactive oxygen species (ROS) (Ross and Siegel, 2017). The NDH complex transfers electrons from LaNDHs via flavin mononucleotide and iron-sulfur centers to quinones within the photosynthetic electron transport chain, and potentially within a chloroplast respiratory chain. Plastoquinone is hypothesized to be the immediate electron acceptor for this enzyme, coupling the redox reaction to proton translocation, which in turn conserves redox energy in the form of a proton gradient. LaNDHs are vital for sustaining the biosynthesis of lavender EOs. However, no studies have yet investigated the specific roles of LaNDHs in *Lavandula angustifolia*.

In this study, we predicted structures using AlphaFold2, and identified their potential active site residues via GalaxyWEB. Gene expression analysis demonstrated that the *LaNDHs* genes (*LaNDH-2*, *LaNDH-11*, *LaNDH-4L1* and *LaNDH-4L2*) exhibited the highest expression levels in leaves compared to other tissues (stems, roots and flowers). Expression of *LaNDHs* in leaves increased with higher cadmium ion (Cd^2+^) concentrations. Additionally, *LaNDHs* expression was elevated as temperature rose from 25 °C to 40 °C and as salt concentrations increased. The highest expression levels of these genes were observed under blue light compared to that under white and red light. Our results suggest that cultivating lavender varieties with enhanced tolerance to abiotic stress could optimize photosynthesis, thereby increasing both the yield and quality of lavender essential oils.

## Results

### Biochemical characteristics of LaNDHs

Bioinformatics analysis of *Lavandula angustifolia* NAD(P)H-quinone oxidoreductases (LaNDHs) was conducted using data obtained from the UniProt database (**Supplementary Table S1**). The molecular weights of these enzymes vary from 11.30 kDa to 84.17 kDa (**Table 1**). The number of amino acids in the LaNDHs proteins ranges from 101 to 739 (**Table 1**). Their isoelectric points (pI) span from 4.19 to 9.53 (**Table 1**). The instability index of these enzymes varies between 22.67 and 55.85 (**Table 1**).

**Table 1:** Physical and chemical properties of LaNDHs.

LaNDHs	Number of amino acids	Molecular formula	Molecular weight (kDa)	Theoretical pI	Instability index
LaNDH-H	393	C_2075_H_3208_N_542_O_572_S_20_	45.54	5.23	35.49
LaNDH-4L1	101	C_511_H_831_N_135_O_142_S_5_	11.30	9.43	35.89
LaNDH-4L2	101	C_510_H_829_N_137_O_142_S_6_	11.34	9.51	31.10
LaNDH-2	510	C_2623_H_4075_N_617_O_712_S_30_	56.61	5.43	41.34
LaNDH-31	120	C_687_H_1016_N_142_O_160_S_4_	13.95	4.73	36.28
LaNDH-J1	158	C_856_H_1282_N_226_O_232_S_5_	18.61	6.58	55.53
LaNDH-32	120	C_686_H_1014_N_142_O_160_S_4_	13.94	4.73	38.59
LaNDH-K	225	C_1137_H_1773_N_301_O_335_S_11_	25.37	8.55	49.36
LaNDH-4	513	C_2749_H_4185_N_623_O_682_S_28_	57.77	7.66	33.11
LaNDH-J2	158	C_855_H_1283_N_225_O_233_S_5_	18.60	6.51	55.85
LaNDH-I1	168	C_872_H_1371_N_237_O_248_S_12_	19.53	8.07	35.62
LaNDH-I2	168	C_870_H_1367_N_235_O_250_S_13_	19.54	7.51	34.02
LaNDH-11	364	C_1925_H_3001_N_447_O_502_S_5_	40.60	5.62	38.96
LaNDH-12	364	C_1927_H_3013_N_447_O_498_S_8_	40.67	8.56	37.46
LaNDH-5	739	C_3968_H_5928_N_938_O_1023_S_32_	84.17	9.17	33.03
LaNDH-61	176	C_917_H_1418_N_200_O_241_S_6_	19.30	4.19	22.67
LaNDH-62	176	C_917_H_1411_N_203_O_241_S_6_	19.33	4.54	25.60

### Secondary structure prediction of LaNDHs

**Figure 1:**
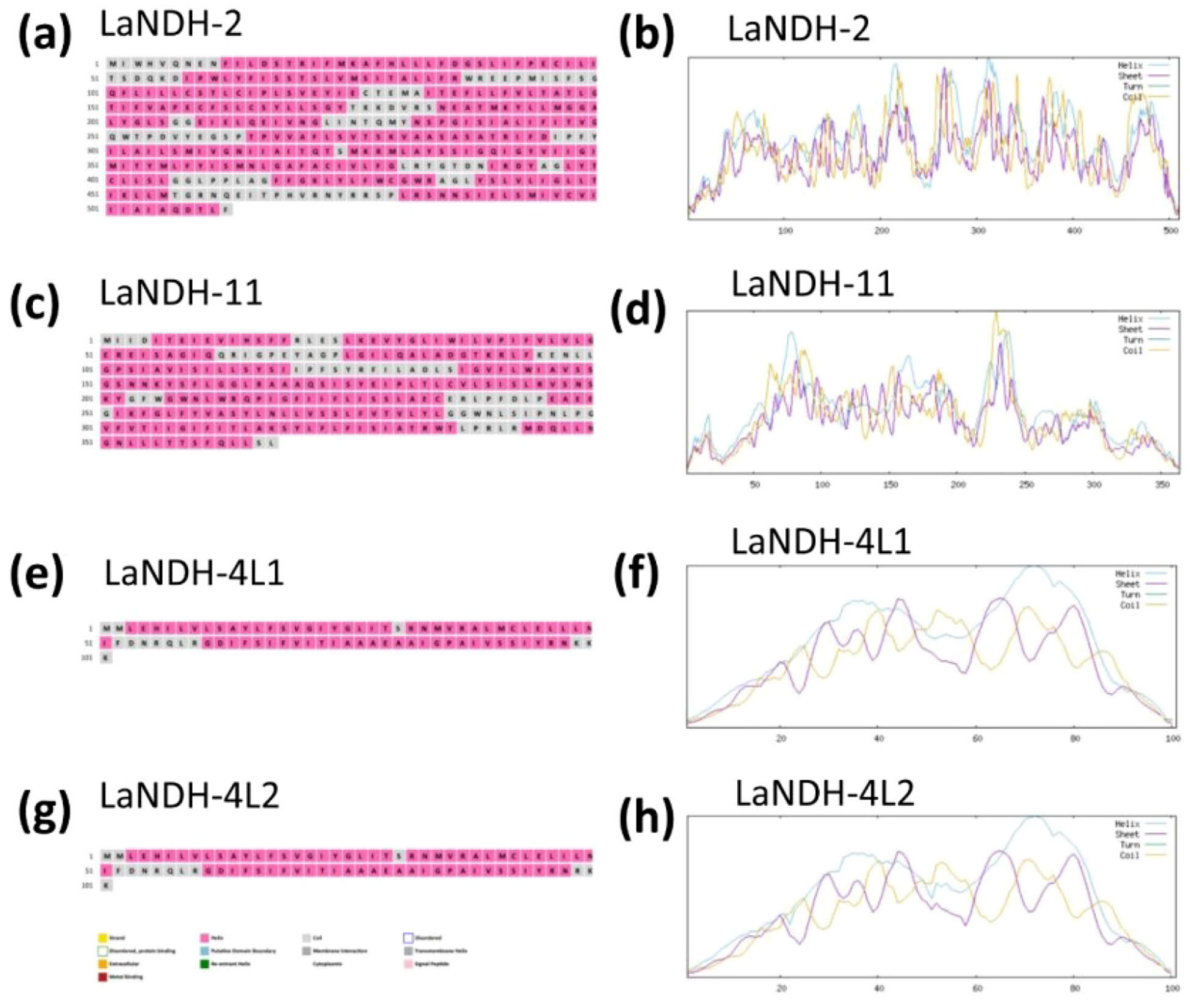
Predicted secondary structure models of **(a, b)** LaNDH-2, **(c, d)** LaNDH-11, **(e, f)** LaNDH-4L1, and **(g, h)** LaNDH-4L2. These secondary structures were predicted using PSIPRED (a for LaNDH-2, c for LaNDH-11, e for LaNDH-4L1, g for LaNDH-4L2) and NPS@ server (b for LaNDH-2, d for LaNDH-11, f for LaNDH-4L1, h for LaNDH-4L2).

Using the amino acid sequences of LaNDH-2, LaNDH-11, LaNDH-4L1 and LaNDH-4L2 (The reasons for our choice of LaNDH-2, LaNDH-11, LaNDH-4L1, and LaNDH-4L2 can be found in the following content.), we predicted their secondary structures using the PSIPRED (Buchan et al., 2024; Jones, 1999) and NPS@ server (Combet et al., 2000) tools, respectively (**Figure 1**; **Tables 1** and **2**). The predicted secondary structures of LaNDH-2, LaNDH-11, LaNDH-4L1, and LaNDH-4L2 are predominantly composed of alpha helices, accounting for 59.02%, 62.36%, 72.28%, and 74.26% of the residues, respectively (**Figure 1**; **Table 1**). Additionally, each protein contains multiple strands and coils (**Figure 1**; **Table 1**). The number of residues in the helices for LaNDH-2, LaNDH-11, LaNDH-4L1, and LaNDH-4L2 are 301, 227, 73, and 75, respectively (**Figure 1**; **Table 1**).

**Table 2:** Secondary structure prediction of LaNDHs.

Secondary structure	Alpha helix		Extended strand		Random coil	
Residual Properties	Number of residues	Total % of residues	Number of residues	Total % of residues	Number of residues	Total % of residues
LaNDH-2	301	59.02	61	11.96	148	29.02
LaNDH-11	227	62.36	35	9.62	102	28.02
LaNDH-4L1	73	72.28	9	8.91	19	18.81
LaNDH-4L2	75	74.26	7	6.93	19	18.81

### Prediction and quality assessment of structural models of LaNDHs

**Figure 2:**
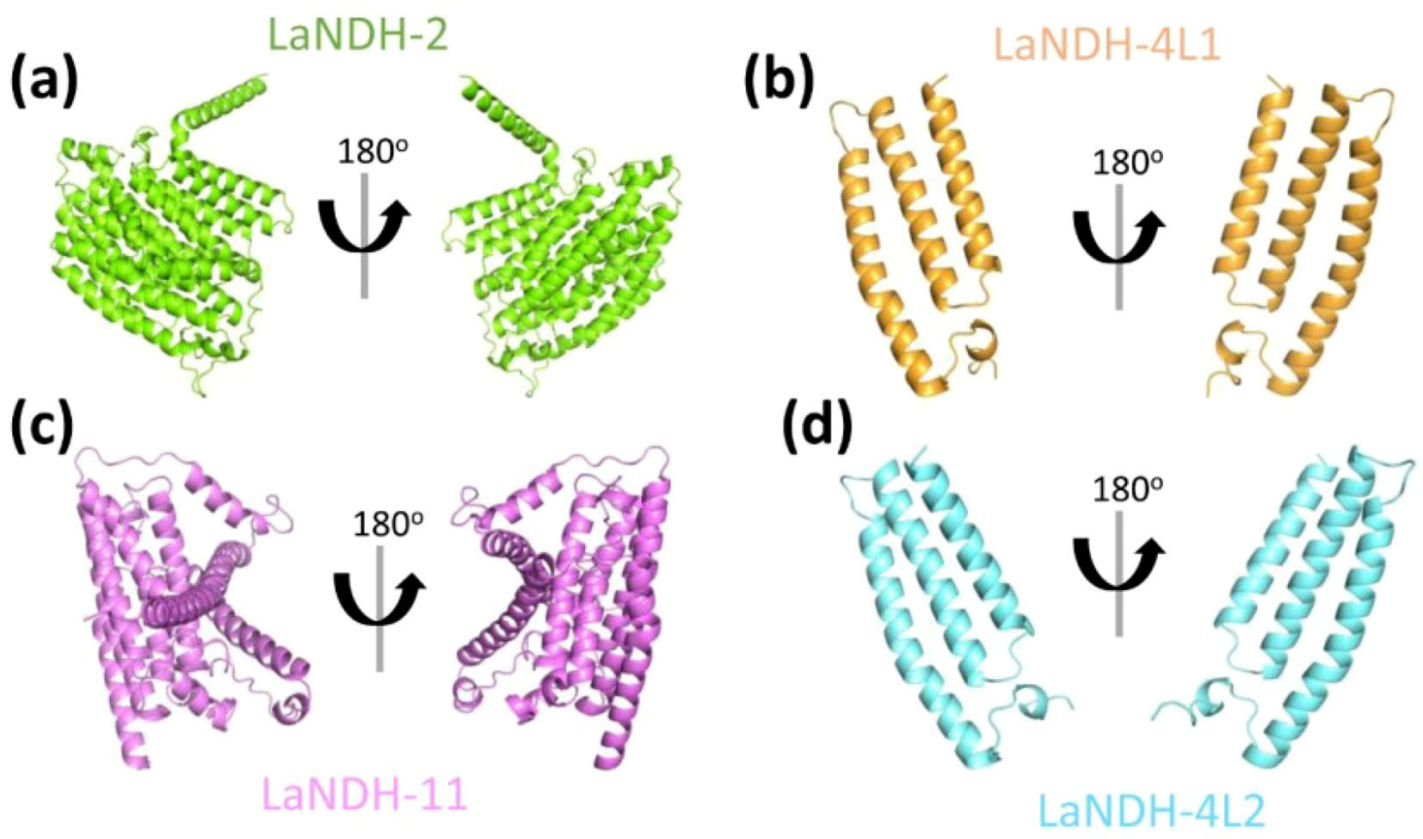
Structural prediction. The three-dimensional (3D) structures of **(a)** LaNDH-2, **(b)** LaNDH-4L1, **(c)** LaNDH-11, and **(d)** LaNDH-4L2 were predicted using AlphaFold2. The predicted structures are shown as ribbon diagrams in two different orientations. The structures of LaNDH-2 **(a)**, LaNDH-4L1 **(b)**, LaNDH-11 **(c)** and LaNDH-4L2 **(d)** are colored in green, orange, magenta and cyan, respectively.

The three-dimensional (3D) structures of LaNDHs were predicted using AlphaFold2 (Wayment-Steele et al., 2023; Jumper et al., 2021). AlphaFold2 is a deep learning-based tool known for providing highly accurate and reliable protein structure predictions, which outperform traditional homology modeling techniques. To assess the quality of the predicted structures (**Figures 2**, **Supplementary Figure S1**), we employed the Ramachandran plot to analyze the dihedral angles of the protein backbones. These ensured they fell within acceptable regions, which indicates a valid protein conformation (**Supplementary Figure S2**; **Table 3**). A high-quality model is expected to have more than 90% of its residues in the most favored regions. In the most favored region, the residual rates of LaNDH-2, LaNDH-11, LaNDH-4L1, and LaNDH-4L2 all exceeded 94%, indicating that these models represent the highest quality structures among these LaNDHs (**Table 3**). Consequently, we proceeded with further analysis using LaNDH-2, LaNDH-11, LaNDH-4L1, and LaNDH-4L2.

**Table 3:** Ramchandran plot analysis of structural models of LaNDHs.

Residues	Residues in most favored regions		Residues in additional allowed regions		Residues in generously allowed regions		Residues in disallowed regions	
Residual Properties	Number of residues	Total % of residues*^a^*	Number of residues	Total % of residues	Number of residues	Total % of residues	Number of residues	Total % of residues
LaNDH-H	299	89.0	34	10.1	2	0.6	1	0.3
LaNDH-4L1	89	94.7	5	5.3	0	0	0	0
LaNDH-4L2	89	94.7	4	4.3	1	1.0	0	0
LaNDH-2	425	94.0	27	6.0	0	0	0	0
LaNDH-31	99	93.4	7	6.6	0	0	0	0
LaNDH-J1	124	90.5	13	9.5	0	0	0	0
LaNDH-32	98	91.6	9	8.4	0	0	0	0
LaNDH-K	164	83.7	29	14.8	3	1.5	0	0
LaNDH-4	421	93.8	27	6.0	1	0.2	0	0
LaNDH-J2	124	90.5	13	9.5	0	0	0	0
LaNDH-I1	139	92.1	10	6.6	1	0.7	1	0.7
LaNDH-I2	140	92.1	11	7.2	1	0.7	0	0
LaNDH-11	301	95.0	15	4.7	0	0	1	0.3
LaNDH-12	300	93.5	19	5.9	1	0.3	1	0.3
LaNDH-5	586	88.4	73	11.0	4	0.6	0	0
LaNDH-61	132	84.6	22	14.1	2	1.3	0	0
LaNDH-62	136	87.2	17	10.9	3	1.9	0	0

*^a^* A good quality model is expected to have over 90% residues in most favored regions.

For LaNDH-2, 94.0% of residues were in the most favored region, 6.0% in the additionally allowed region, and none in the generously allowed or disallowed regions (**Table 3**). For LaNDH-11, 95.0% of residues were in the most favored region, 4.7% in the additionally allowed region, 0.3% in the disallowed region, and none in the generously allowed region (**Table 3**). For LaNDH-4L1, 94.7% of residues were in the most favored region, 5.3% in the additionally allowed region, and none in the generously allowed or disallowed regions (**Table 3**). For LaNDH-4L2, 94.7% of residues were in the most favored region, 4.3% in the additionally allowed region, 1.0% in the generously allowed region, and none in the disallowed region (**Table 3**).

**Figure 3:**
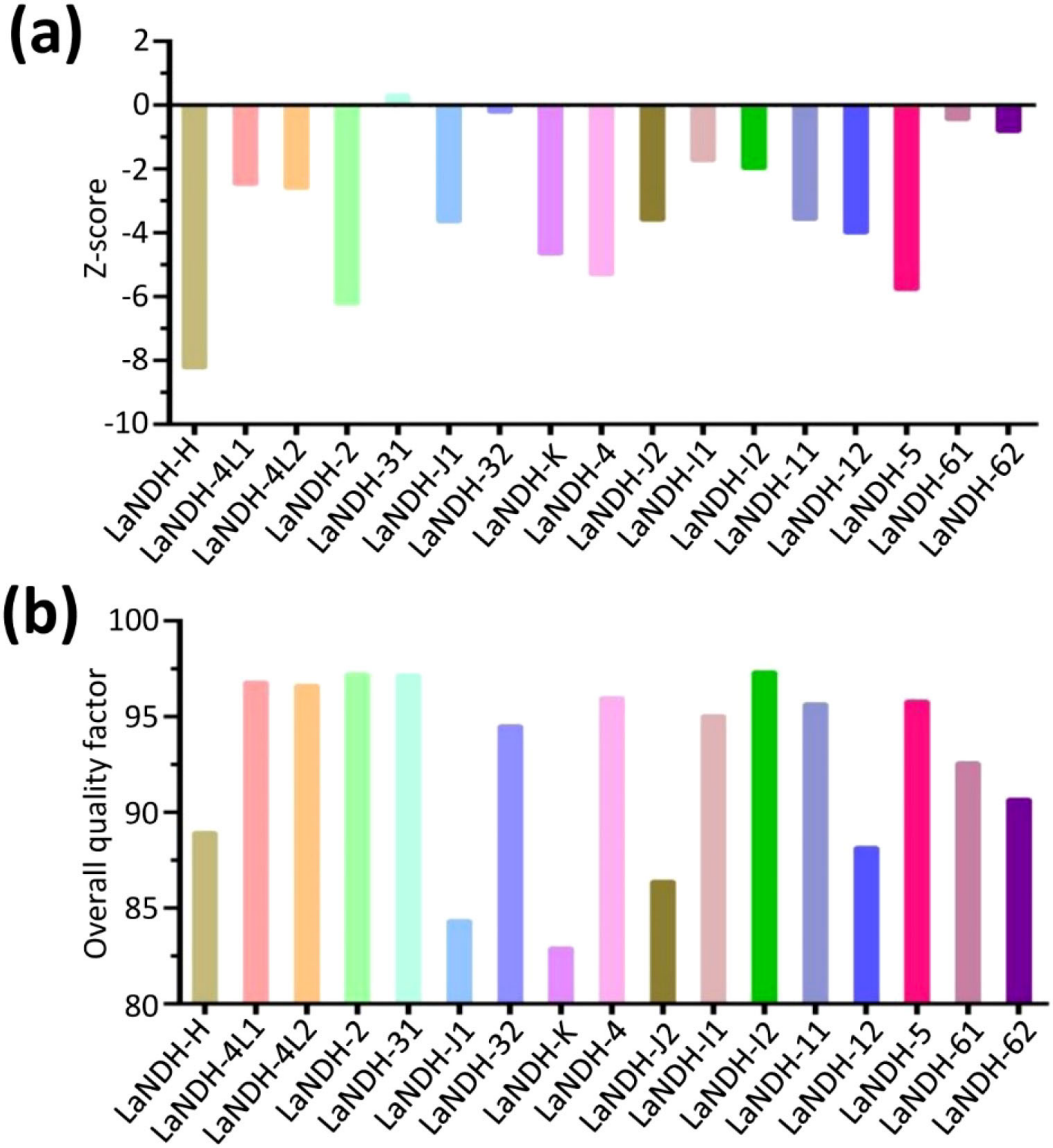
Structural quality assessment. **(a)** The reliability of the predicted models was assessed using ProSA, which calculated Z-scores to evaluate the global quality of the models. **(b)** Furthermore, the overall quality factor was determined to further confirm the structural integrity of the models.

ProSA analysis of the models revealed Z-scores of -6.22, -3.58, -2.47, and -2.59 for LaNDH-2, LaNDH-11, LaNDH-4L1, and LaNDH-4L2, respectively (**Figures 3a**, **Supplementary Figure S3**). The overall quality factors of these models were 97.21, 95.66, 96.63, and 96.63, respectively (**Figure 3b**), further confirming the high quality of the predicted structures.

### Predicting active sites of LaNDHs

**Figure 4:**
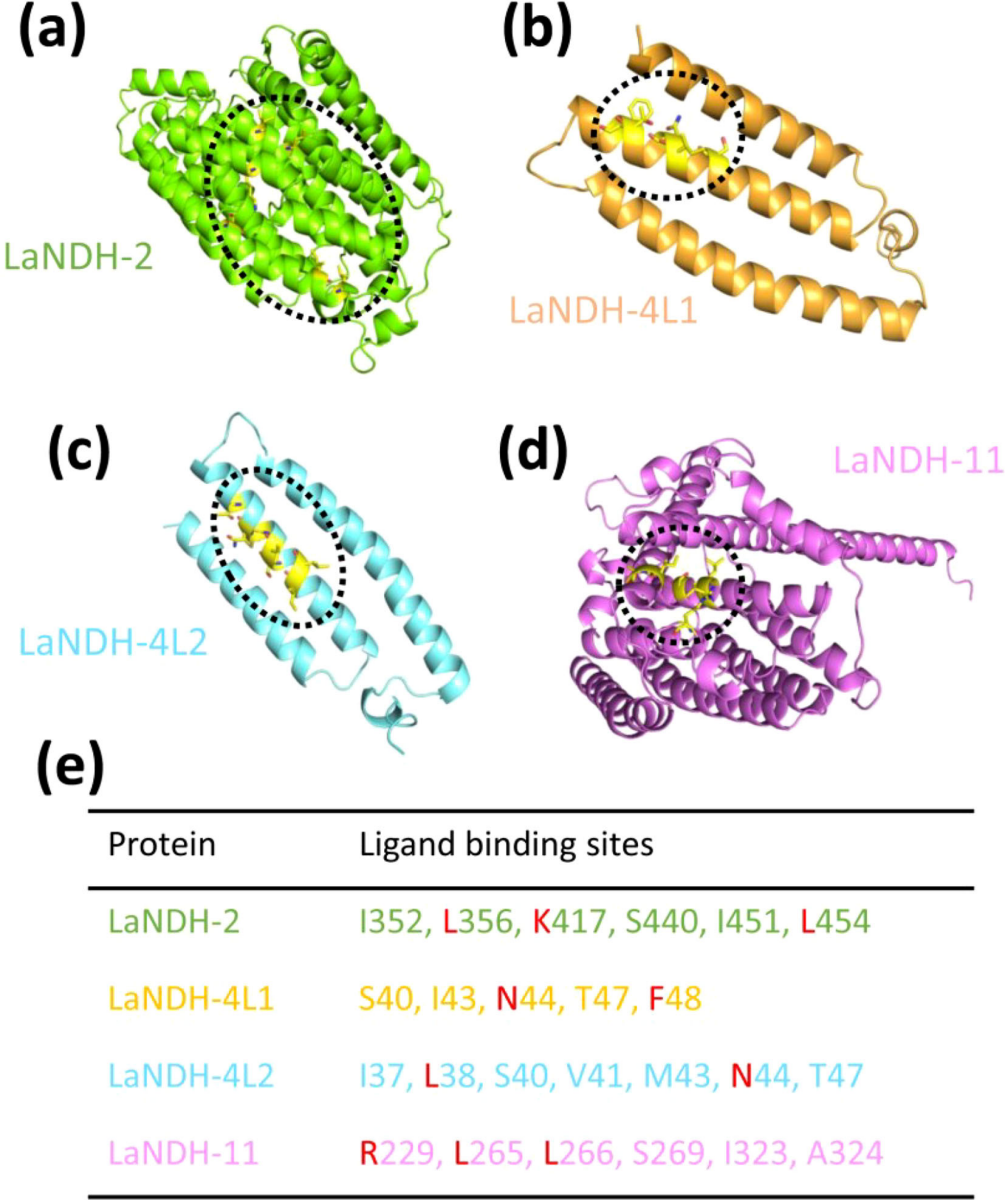
Predicting **(a)** LaNDH-2, **(b)** LaNDH-4L1, **(c)** LaNDH-4L2 and **(d)** LaNDH-11 active site residues using the GalaxyWEB program. **(e)** The residues in the active site of LaNDH-2, LaNDH-4L1, LaNDH-4L2 and LaNDH-11. The residues (L, K, N, F and R) marked in red are evolutionarily conserved among plant NAD(P)H-quinone oxidoreductases.

Using the predicted models (**Figure 2**), we employed the GalaxyWEB program (Ko et al., 2012; Heo et al., 2013, 2016; Seok et al., 2021) to identify the active sites of LaNDH-2, LaNDH-11, LaNDH-4L1, and LaNDH-4L2 (**Figure 4**). The results revealed that the active site residues of LaNDH-2 include I352, L356, K417, S440, I451, and L454 (**Figures 4a, e**). For LaNDH-4L1, the active site residues were identified as S40, I43, N44, T47, and F48 (**Figures 4b, e**). For LaNDH-4L2, the active site residues include I37, L38, S40, V41, M43, N44, and T47 (**Figures 4c, e**). In the case of LaNDH-11, the active site residues consist of R229, L265, L266, S269, I323, and A324 (**Figures 4d, e**). These residues are highly likely to be involved in the catalytic process, potentially interacting with the substrate side chain atoms to form essential bonds.

### Gene *LaNDHs* exhibit the highest expression level in leaves among lavender tissues

**Figure 5:**
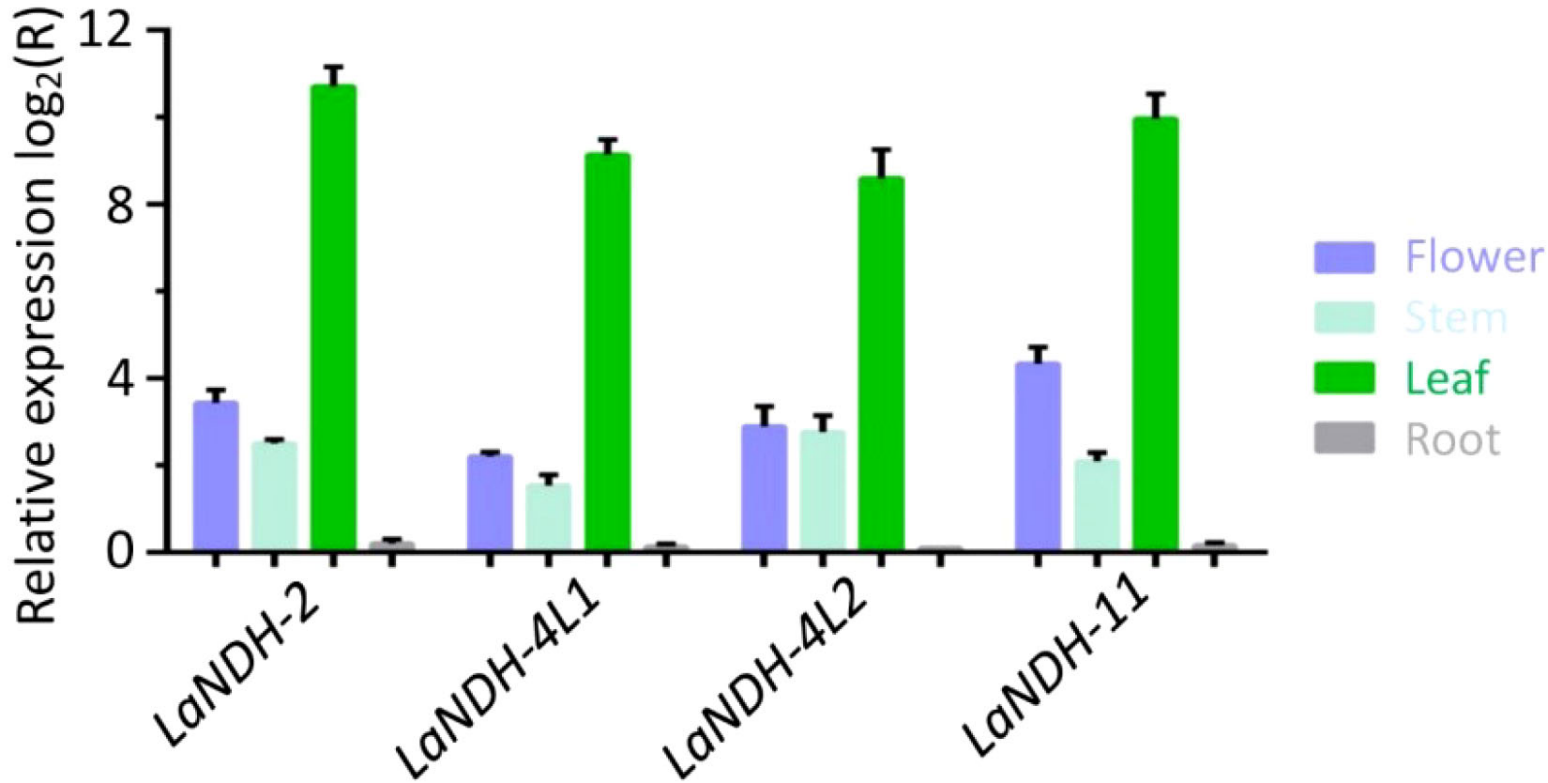
Gene expression levels in different tissues (root, stem, leaf, and flower) using reverse transcription quantitative PCR (RT-qPCR). Comparative analysis showed a marked increase in gene expression in leaf tissue compared to root, stem, and floral tissues. Gene expression was quantitatively assessed using the 2^-ΔΔCT^ method, with beta-actin serving as the reference gene.

To examine the expression profiles of LaNDH-2, LaNDH-11, LaNDH-4L1, and LaNDH-4L2 across various tissues (leaves, stems, flowers, and roots), we conducted real-time quantitative polymerase chain reaction (RT-qPCR). The results indicated that the highest expression levels of *LaNDH-2*, *LaNDH-11*, *LaNDH-4L1*, and *LaNDH-4L2* were found in the leaves compared to other tissues (**Figure 5**). Specifically, the expression of *LaNDH-2* was upregulated by 1663.5-fold in leaves, 10.6-fold in flowers, 5.7-fold in stems, and 1.1-fold in roots (**Figure 5**). *LaNDH-11* expression was increased by 560.3-fold in leaves, 4.6-fold in flowers, 2.9-fold in stems, and 1.1-fold in roots (**Figure 5**). For *LaNDH-4L1*, expression was upregulated by 388.0-fold in leaves, 7.5-fold in flowers, 6.8-fold in stems, and 1.1-fold in roots (**Figure 5**). *LaNDH-4L2* expression increased by 812.9-fold in leaves, 20.2-fold in flowers, 4.3-fold in stems, and 1.2-fold in roots (**Figure 5**). These results suggest that *LaNDH-2*, *LaNDH-11*, *LaNDH-4L1*, and *LaNDH-4L2* are predominantly expressed in leaf tissue, implying their primary involvement in chloroplast-based photosynthetic processes.

### Expression profiles of gene *LaNDHs* under different abiotic stress conditions

**Figure 6:**
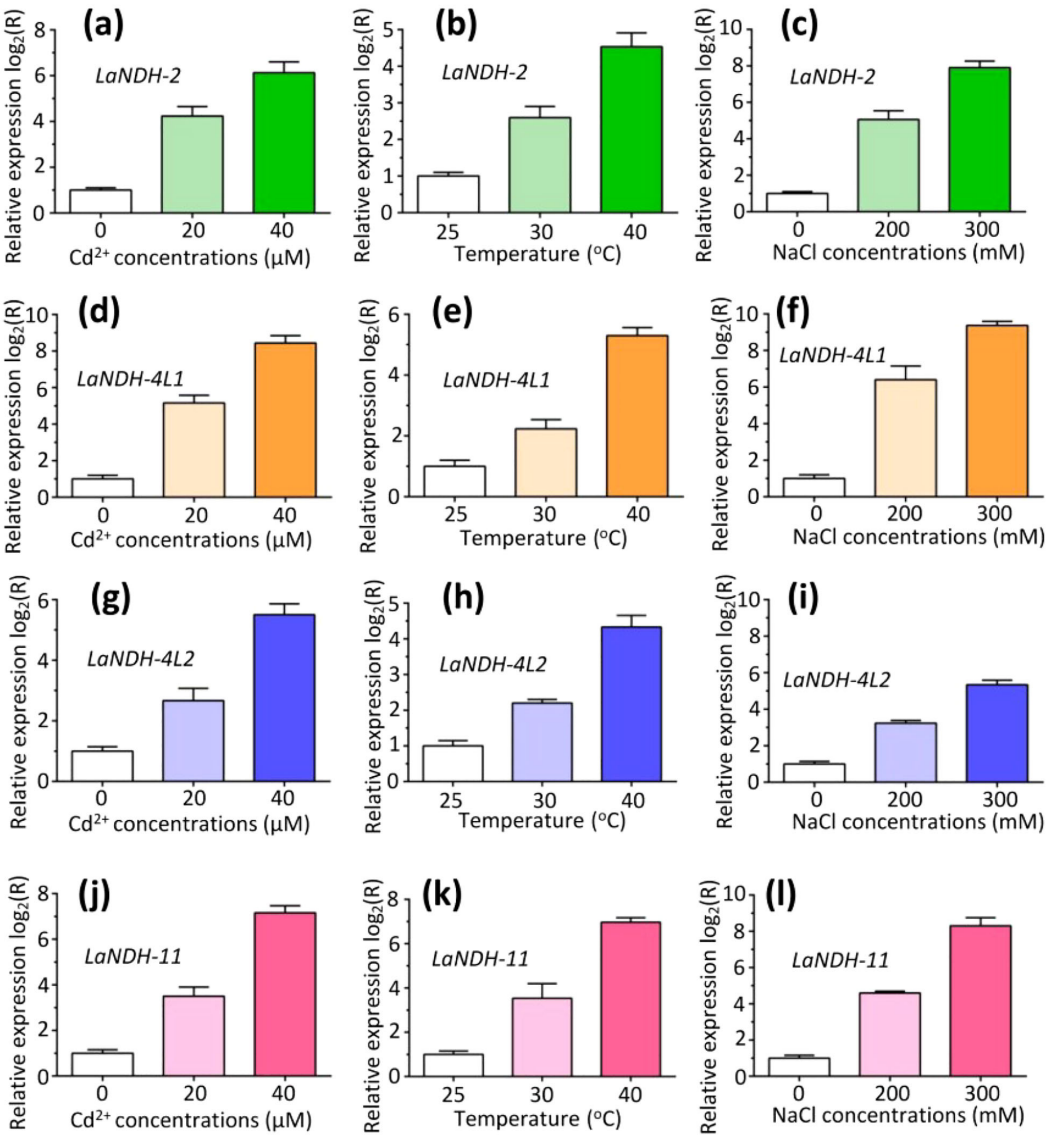
The expression profiles of genes **(a-c)** *LaNDH-2*, **(d-f)** *LaNDH-4L1*, **(g-i)** *LaNDH-4L2*, and **(j-l)** *LaNDH-11* in leaf under abiotic stress conditions, including cadmium ion (Cd^2+^), heat, and NaCl exposure. For Cd^2+^ stress **(a, d, g, j)**, plants were subjected to 0, 20, and 40 µM Cd^2+^ treatments. Heat stress **(b, e, h, k)** involved exposure to temperatures of 25°C, 30°C, and 40°C, respectively. Salt stress **(c, f, i, l)** was applied using 0, 200, and 300 mM NaCl treatments. Relative gene expression was quantified by RT-qPCR, with untreated samples normalized to a baseline value of 1.

We performed RT-qPCR analysis to assess the expression levels of *LaNDHs* (*LaNDH-2*, *LaNDH-11*, *LaNDH-4L1*, and *LaNDH-4L2*) in response to cadmium ion (Cd^2+^), heat, and salt treatments in leaves (**Figure 6**). The results revealed that the expression of *LaNDHs* in leaves was positively correlated with increasing Cd^2+^ concentrations (**Figures 6a, d, g, j**). Similarly, *LaNDHs* expression in leaves increased as the temperature rose from 25°C to 40°C (**Figures 6b, e, h, k**). Additionally, *LaNDHs* expression in leaves was upregulated with higher salt concentrations (**Figures 6c, f, i, l**). These findings suggest that cadmium ion, heat and salt stress influence the photosynthetic rate in lavender, providing evidence for the association between *LaNDHs* genes (*LaNDH-2*, *LaNDH-11*, *LaNDH-4L1*, and *LaNDH-4L2*) and the photosynthetic process.

### Differential expression of gene *LaNDHs* under various light conditions

**Figure 7:**
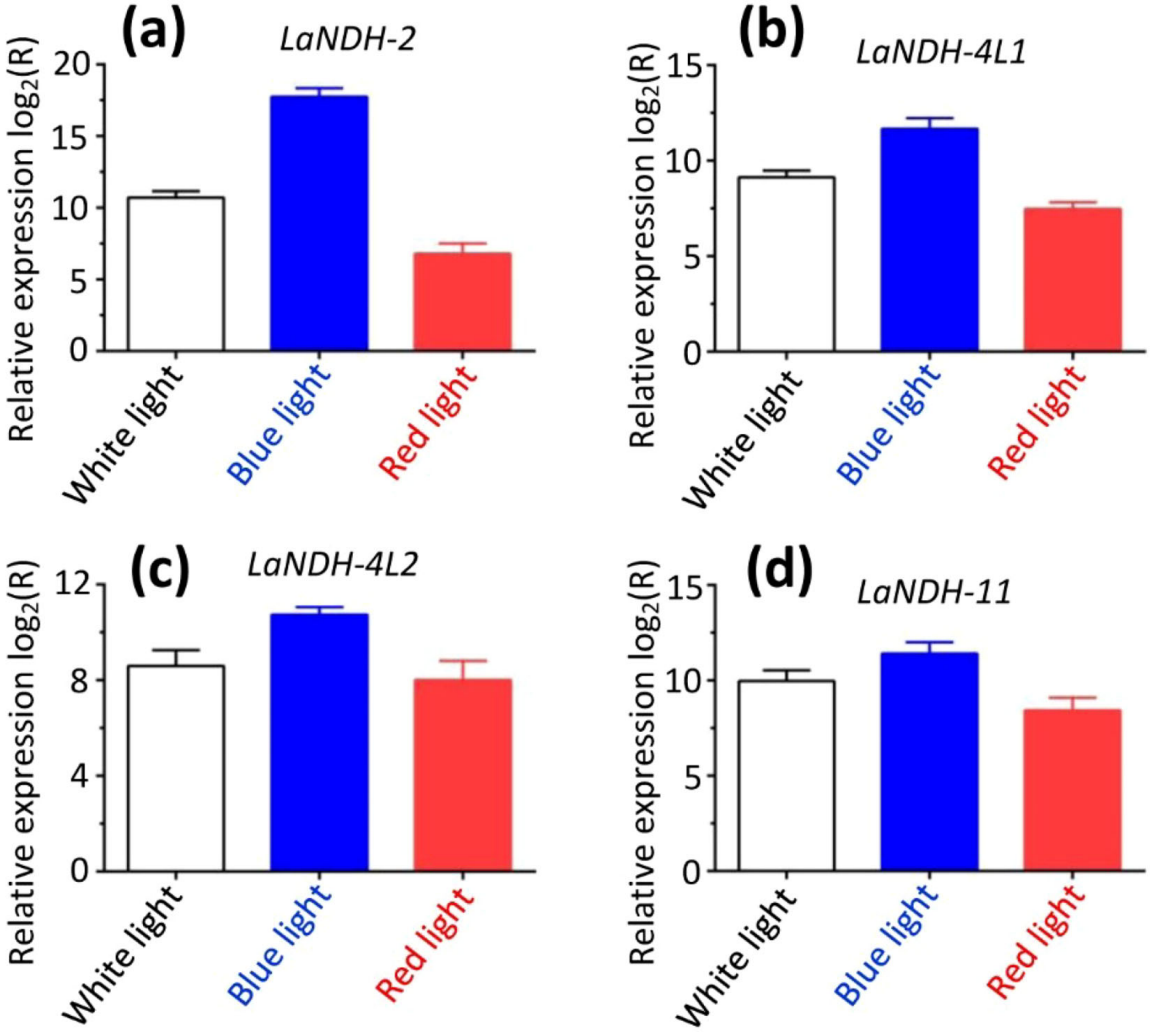
Gene expression in leaf under different light conditions (white, blue, and red light). Among the various treatments, blue light resulted in the highest expression levels of the genes **(a)** *LaNDH-2*, **(b)** *LaNDH-4L1*, **(c)** *LaNDH-4L2*, and **(d)** *LaNDH-11*. Relative gene expression was quantified by RT-qPCR, with beta-actin serving as the reference gene. Data analysis was performed using the 2^-ΔΔCT^ method.

To establish a comprehensive light-responsive gene expression profile, we evaluated the expression levels of *LaNDHs* genes (*LaNDH-2*, *LaNDH-11*, *LaNDH-4L1*, and *LaNDH-4L2*) in leaves under various light conditions (white, red, and blue) using RT-qPCR. The results showed that the expression levels of these genes were highest under blue light compared to other light conditions (**Figure 7**). Specifically, for *LaNDH-2*, the expression was highest under blue light (217,898.9-fold), followed by white light (1,663.5-fold), and red light (111.4-fold) (**Figure 7a**). For *LaNDH-4L1*, the highest expression was observed under blue light (3,251.1-fold), followed by white light (561.6-fold), and red light (176.9-fold) (**Figure 7b**). For *LaNDH-4L2*, the expression peaked under blue light (1,702.3-fold), followed by white light (388.1-fold), and red light (256.3-fold) (**Figure 7c**). For *LaNDH-11*, the highest expression was found under blue light (2,786.4-fold), followed by white light (812.4-fold), and red light (345.7-fold) (**Figure 7d**). These findings underscore the significant role of light in regulating the expression of *LaNDHs* genes (*LaNDH-2*, *LaNDH-11*, *LaNDH-4L1*, and *LaNDH-4L2*), further supporting the connection between these genes and photosynthesis.

## Discussion

In this work, we used PSIPRED and NPS@ server to predict the secondary structures of LaNDH-2, LaNDH-11, LaNDH-4L1, and LaNDH-4L2, and their structural models were generated with AlphaFold2. The GalaxyWEB program was then applied to identify potential active site residues for these proteins. Gene expression analysis showed that the *LaNDHs* genes (*LaNDH-2*, *LaNDH-11*, *LaNDH-4L1*, and *LaNDH-4L2*) were most highly expressed in the leaves compared to other tissues (stems, roots, and flowers). Expression levels of *LaNDHs* in leaves increased with higher cadmium ion (Cd^2+^) concentrations. Additionally, *LaNDHs* expression in leaves rose as the temperature increased from 25 °C to 40 °C and with higher salt concentrations. Among different light conditions (white, blue, and red), the expression levels of *LaNDHs* genes were highest under blue light. Given their localization in the chloroplast, these genes may be involved in lavender photosynthesis. LaNDH-4L1/4L2 could be targets for stress-tolerant lavender varieties. These findings suggest that cultivating lavender varieties tolerant to abiotic stress could enhance photosynthetic efficiency, thereby improving both the yield and quality of lavender essential oils (EOs).

LaNDHs may confer enhanced stress tolerance through multifaceted mechanisms. Functioning as a pivotal enzyme in redox homeostasis, LaNDHs mitigate oxidative damage by facilitating electron transfer from NAD(P)H to quinones, thereby scavenging reactive oxygen species (ROS). LaNDHs potentially contribute to cyclic electron flow around Photosystem I, optimizing ATP/NADPH ratios and alleviating photo-oxidative stress. Notably, blue light specifically induces *LaNDHs* upregulation, likely mediated by specialized photoreceptors or chloroplast-derived signaling cascades. Therefore, LaNDH represents a promising genetic target for enhancing lavender stress adaptability. Potential breeding applications of LaNDHs include: (1) Genetic engineering - overexpressing LaNDH via CRISPR-Cas9 or stress-responsive promoters to bolster drought and salinity tolerance; (2) Pre-transplant conditioning - using blue light priming to pre-activate LaNDH expression in seedlings prior to field transplantation. These strategies could enhance lavender resilience to stress without compromising the yield or quality of its EOs.

Photosynthesis is the fundamental physiological and biochemical process on Earth, underpinning plant growth, development, and the production of high yield and quality. Over ninety percent of a plant dry mass is derived from products of leaf photosynthesis (Hagemann and Bauwe, 2016; Johnson, 2016; Silveira and Carvalho, 2016). Various factors influence the efficiency of photosynthesis: Light provides the necessary energy, with its intensity and wavelength directly affecting the rate of photosynthesis. Carbon dioxide is crucial for the Calvin cycle, acting as a limiting factor when present at low concentrations (von Caemmerer and Furbank, 2016; Szechyńska-Hebda et al., 2017; Dusenge et al., 2018; Sekhar et al., 2020). Temperature impacts enzyme function, with optimal conditions typically ranging between 20–30 °C. Water availability is essential for maintaining turgor pressure and facilitating stomatal opening for gas exchange. Chlorophyll content governs the plant ability to absorb light. Additionally, oxygen competes with carbon dioxide during photorespiration, reducing yields in C3 plants. Plant adaptations, such as C4 and Crassulacean acid metabolism (CAM) pathways, along with leaf anatomical features, also play significant roles (von Caemmerer and Furbank, 2016; Sekhar et al., 2020; Cruz and Avenson, 2021; Guirguis et al., 2023). The overall photosynthetic rate is ultimately constrained by the slowest limiting factor.

Current research on the impact of abiotic stress on photosynthesis in lavender has primarily concentrated on drought stress, which inhibits growth and reduces photosynthetic pigment levels. These findings provide a theoretical foundation for the cultivation and industrialization of lavender in environments subject to stress (Li et al., 2023; Croce et al., 2024; Marulanda Valencia and Pandit, 2024; Shomali et al., 2024). Lavender typically thrives in temperatures ranging from 15 °C to 30 °C. Other previous studies have demonstrated that exposure to low-temperature stress (0 °C) can activate the expression of genes involved in the synthesis of protective compounds, such as fatty acid desaturases and soluble sugars, which contribute to the formation of a cold signaling regulatory network (Li et al., 2023; Croce et al., 2024; Marulanda Valencia and Pandit, 2024; Shomali et al., 2024). This network ultimately enhances lavender cold tolerance.

In summary, our study introduces a new approach to thoroughly investigate the functional mechanisms of NAD(P)H-quinone oxidoreductases in *Lavandula angustifolia*, with the objective of increasing the yield and enhancing the quality of lavender essential oils (EOs).

## Materials and methods

### Bioinformatics analysis

The amino acid sequences of *Lavandula angustifolia* NAD(P)H-quinone oxidoreductases (LaNDHs) (**Supplementary Table S1**) were analyzed using ProtParam to predict their chemical properties and physicochemical parameters (Duvaud et al., 2021; Gasteiger, 2003).

### Prediction of structural models

Secondary structures were predicted using PSIPRED 4.0 (Buchan et al., 2024; Jones, 1999) and the NPS@ v2.16.0 (Combet et al., 2000). Three-dimensional structural predictions for LaNDHs were carried out with the AlphaFold2 v2.1.1 (Wayment-Steele et al., 2023; Jumper et al., 2021). Active site residues were identified using the GalaxyWEB program (Ko et al., 2012; Heo et al., 2013, 2016; Seok et al., 2021). Multiple sequence alignment was performed using the LSQKAB program within the CCP4 suite (Collaborative Computational Project N, 1994), and the root mean square deviation (RMSD) for Cα atoms was calculated. Structural visualizations were generated using PyMOL 2.3.4 (https://www.pymol.org/2/).

### Quality assessment of structural models of LaNDHs

To validate the tertiary structures, Ramachandran plots for LaNDHs were generated using the PDBsum database (de Beer et al., 2014; Laskowski et al., 2017; Laskowski, 2022, 2004). This tool evaluates the quality of protein structures by detecting geometric errors, thereby enhancing the accuracy of the models. The Ramachandran plot specifically analyzes the stereochemical properties by displaying the dihedral angles of amino acid residues, identifying the allowed conformational regions, and highlighting any disallowed orientations.

On the other hand, ProSA (Protein Structure Analysis) is a commonly used tool for analyzing and validating predicted protein models (Wiederstein and Sippl, 2007). The z-score provides an overall assessment of model quality and is plotted against the z-scores of all experimentally determined protein structures in the current PDB. This plot distinguishes between structural types (e.g., X-ray, NMR) using color coding, enabling the evaluation of whether the z-score for the input structure falls within the expected range for native proteins of similar size.

### Analysis of gene expression levels of *LaNDHs* using RT-qPCR

To quantify the expression levels of the target gene under different light conditions, real-time quantitative polymerase chain reaction (RT-qPCR) was conducted using PowerUp SYBR Green Master Mix (Applied Biosystems). Plant tissue samples (roots, stems, leaves, and flowers) were collected, immediately flash-frozen in liquid nitrogen, and stored at -80°C for later analysis. The light treatments included white, red, and blue light, with red light having a maximum wavelength of 660 nm and blue light having a maximum wavelength of 450 nm. The light intensity was set at 100 µmol/(m·s). For cadmium ion (Cd^2+^) stress, concentrations of 0, 20, and 40 µM Cd^2+^ were applied, while temperature stress was tested at 25°C, 30°C, and 40°C. Salt stress was induced using 0, 200, and 300 mM NaCl, respectively. Total RNA was extracted using the Universal Plant Total RNA Extraction Kit (Bioteke, Beijing, China) according to the manufacturer’s protocol. cDNA synthesis was performed with the PrimeScript 1st Strand cDNA Synthesis Kit (Takara, Kyoto, Japan). Primer sequences are listed in **Supplementary Table S2**. The PCR reaction volume was 20 μL, with the following conditions: 90°C for 5 min, followed by 40 cycles of 95°C for 10 s and 60°C for 30 s, and a final step of 95°C for 15 s and 60°C for 60 s. RT-qPCR was performed using an Applied Biosystems QuantStudio 5 instrument. Data were analyzed using the 2^-ΔΔCT^ method (Hawkins and Guest, 2017; Green and Sambrook, 2018), and relative expression levels were presented as log_2_ values in histograms. Beta-actin gene is expressed at relatively constant levels in different tissues and cells and is used to detect changes in gene expression levels. Beta-actin was used as the reference gene, with expression normalized to untreated controls. A positive control was included for the beta-actin gene. A ratio greater than zero indicated upregulation, while a ratio less than zero indicated downregulation.

### Statistical analysis

All experiments were conducted at least in triplicate. The data were expressed as mean ± SD. Statistical analysis was conducted using Origin 8.5, Microsoft Excel 2013 and SPSS 19.0. In the all statistical evaluations, *p* < 0.05 was considered statistically significant, and *p* < 0.01 was considered high statistically significant.

## Data Availability

The original contributions presented in the study are included in the article/**Supplementary Material**. Further inquiries can be directed to the corresponding author.
