# Supplementary material for: Structural and functional insights into NAD(P)H-quinone oxidoreductases in lavender: implications for abiotic stress tolerance and essential oil production
Source: Front Plant Sci. 2025 Aug 28;16:1661227. doi: 10.3389/fpls.2025.1661227 (PMC12423422; doi:10.3389/fpls.2025.1661227)
Supplement: Supplementary file 1 [file DataSheet1.docx]

**Supplementary Material**

**Structural and Functional Insights into NAD(P)H-Quinone Oxidoreductases in Lavender: Implications for Abiotic Stress Tolerance and Essential Oil Production**

Dafeng Liu^1,3,*^, Na Li^1,3^, Huashui Deng^2^, Daoqi Song^2^, Minawaier Maimaiti^1^, Ayidana Nuerbieke^1^, Mingtai Yekepeng^1^ and Kailibinuer Aili^1^

^1^Xinjiang Key Laboratory of Lavender Conservation and Utilization, College of Biological Sciences and Technology, Yili Normal University, Yining 835000, Xinjiang, China;

^2^School of Life Sciences, Xiamen University, Xiamen 361102, Fujian, China;

^3^These authors contributed equally to the article.

^*^Correspondence: dafeli@sina.cn or dafeli-dafeli@hotmail.com

**Table S1.**

**Table S1. LaNDHs from UniProt database**

| LaNDHs | UniProt ID | Full names | Genes |
| --- | --- | --- | --- |
| LaNDH-H | A0A125QY07 | NAD(P)H-quinone oxidoreductase subunit H | *ndhH* |
| LaNDH-4L1 | A0A125QY08 | NAD(P)H-quinone oxidoreductase subunit 4L | *ndhE* |
| LaNDH-4L2 | A0A2R2V084 | NAD(P)H-quinone oxidoreductase subunit 4L | *ndhE* |
| LaNDH-2 | A0A0X9RI43 | NAD(P)H-quinone oxidoreductase subunit 2 | *ndhB* |
| LaNDH-31 | A0A2R2V061 | NAD(P)H-quinone oxidoreductase subunit 3 | *ndhC* |
| LaNDH-J1 | A0A2R2V063 | NAD(P)H-quinone oxidoreductase subunit J | *ndhJ* |
| LaNDH-32 | A0A109PQI5 | NAD(P)H-quinone oxidoreductase subunit 3 | *ndhC* |
| LaNDH-K | A0A109PTJ0 | NAD(P)H-quinone oxidoreductase subunit K | *ndhK* |
| LaNDH-4 | A0A0X9SIU7 | NAD(P)H-quinone oxidoreductase chain 4 | *ndhD* |
| LaNDH-J2 | A0A0X9SIW9 | NAD(P)H-quinone oxidoreductase subunit J | *ndhJ* |
| LaNDH-I1 | A0A109PQS5 | NAD(P)H-quinone oxidoreductase subunit I | *ndhI* |
| LaNDH-I2 | A0A2R2V096 | NAD(P)H-quinone oxidoreductase subunit I | *ndhI* |
| LaNDH-11 | A0A2R2V095 | NAD(P)H-quinone oxidoreductase subunit 1 | *ndhA* |
| LaNDH-12 | A0A125QY03 | NAD(P)H-quinone oxidoreductase subunit 1 | *ndhA* |
| LaNDH-5 | A0A0X9UAN1 | NAD(P)H-quinone oxidoreductase subunit 5 | *ndhF* |
| LaNDH-61 | A0A2R2V081 | NAD(P)H-quinone oxidoreductase subunit 6 | *ndhG* |
| LaNDH-62 | A0A109PM26 | NAD(P)H-quinone oxidoreductase subunit 6 | *ndhG* |

**Table S2.**

**Table S2. Primers used for RT-qPCR in this study**

| Genes | Primers | Primer sequence (5’-3’) |
| --- | --- | --- |
| *Beta-actin* | Forward primer | ggcagtttggacaagagaagacacagtcacc |
|  | Reverse primer | ttttttgattaaaaaaaaaagctgaaatcataatatttttaat |
| *LaNDH-2* | Forward primer | atgatctggcatgtaagaatgaaaacttcattctcgattctac |
|  | Reverse primer | ctaaaaaagggtatcctgagcaattgcaataatcgggttc |
| *LaNDH-4L1* | Forward primer | tcctggaggaatatgtgcatctaaaagatcctt |
|  | Reverse primer | atgctcacgatttcgatagccatac |
| *LaNDH-4L2* | Forward primer | aaatagaatttctttcttcgtctttacaaaaaaaaaaaaata |
|  | Reverse primer | attgtcgaaattccgttttctctacaattacta |
| *LaNDH-11* | Forward primer | aaatagaatttctttcttcgtctttacaaaaaaaaaaaaataggag |
|  | Reverse primer | gaaattcaaatggagaaaatccaaagatatttacaattacaga |

**Figure S1.**


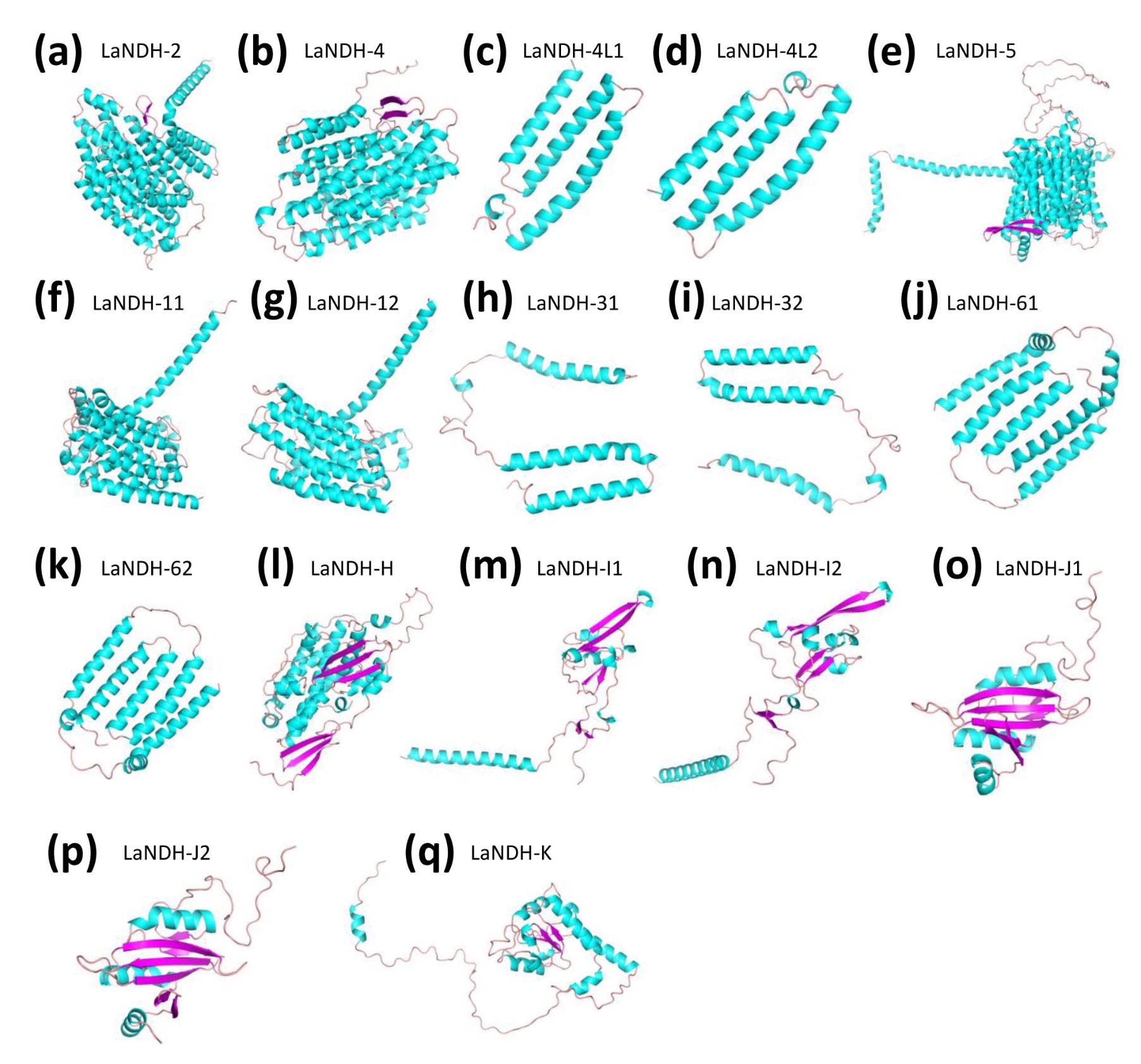


**Figure S1. The predicted three-dimensional (3D) structural models are depicted in cyan ribbon representation from two perspectives, with alpha-helices highlighted in pink and beta-sheets in cyan.** These models were generated using AlphaFold2.

**Figure S2.**


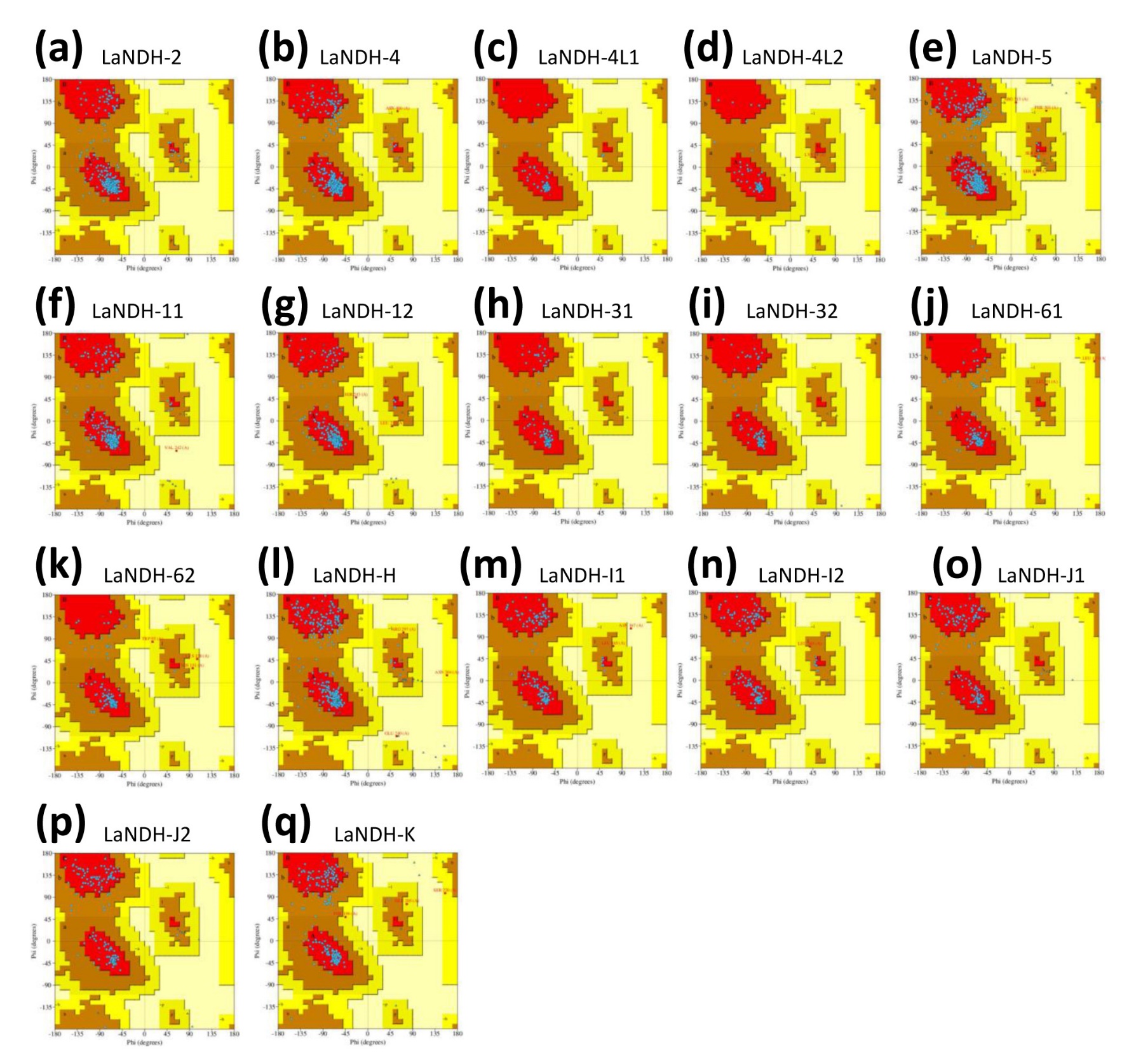


**Figure S2. Structural validation was performed using Ramachandran plot analysis, with the most favorable conformational regions indicated in red and less favorable regions depicted in progressively lighter shades.**

**Figure S3.**


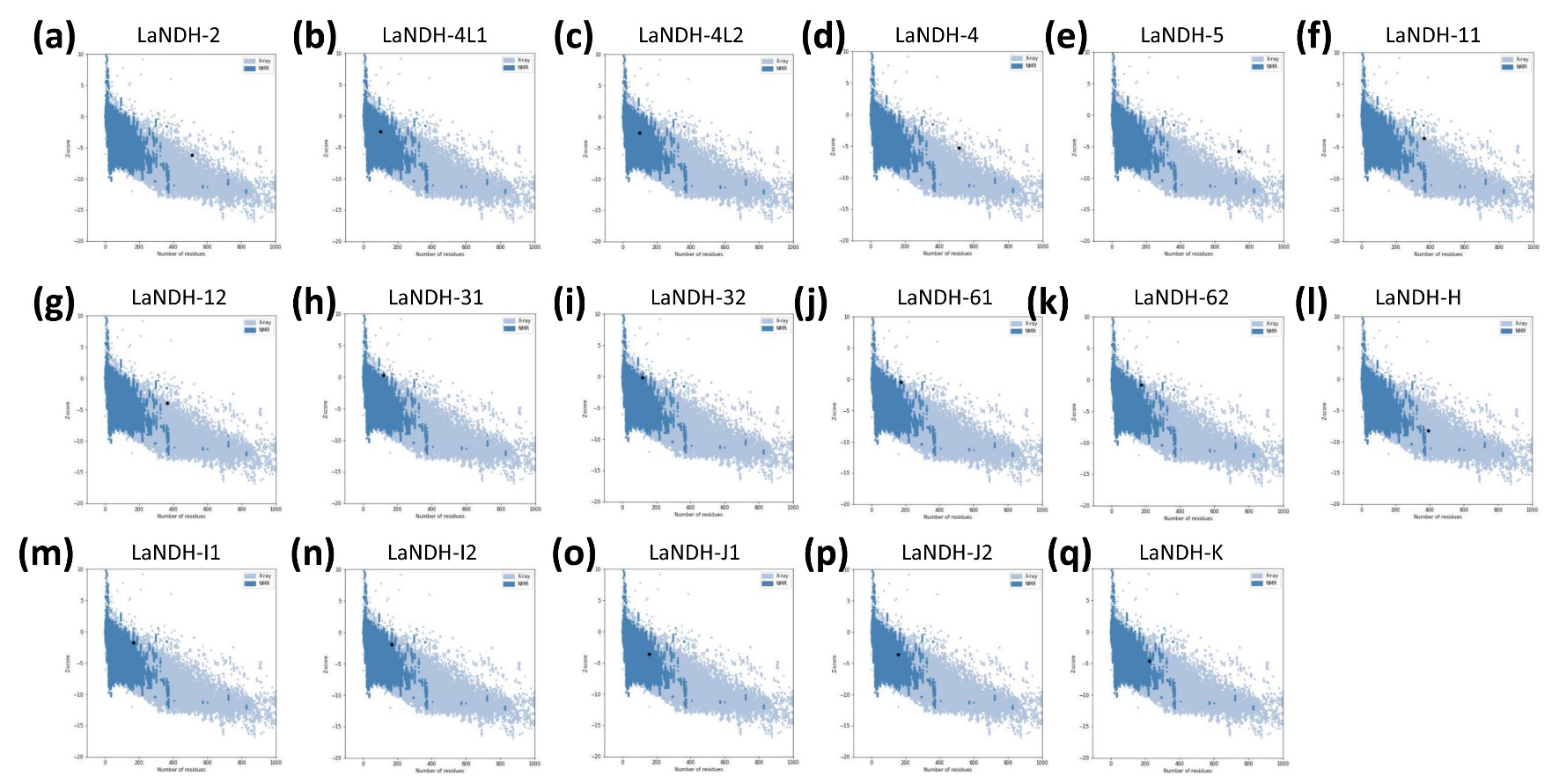


**Figure S3. Structural validation was conducted via ProSA analysis.**
